# Factors associated with musculoskeletal symptoms and heart rate variability among cleaners – cross-sectional study

**DOI:** 10.1186/s12889-020-08928-7

**Published:** 2020-05-24

**Authors:** Josiane Sotrate Gonçalves, Tatiana de Oliveira Sato

**Affiliations:** Physical Therapy Department, Laboratory of Preventive Physical Therapy and Ergonomics (LAFIPE), Physical Therapy Postgraduate Program, Federal University of São Carlos, Rodovia Washington Luís, km 235, Monjolinho, São Carlos, SP 13565-905 Brazil

**Keywords:** Ergonomics, Cleaners, Work-related musculoskeletal disorders, Cardiac autonomic modulation

## Abstract

**Background:**

The professionals who perform cleaning activity constitute a major economic sector in Brazil. Cleaners may develop health problems related to the musculoskeletal and cardiovascular systems. It is necessary to understand the working and health conditions of cleaners in Brazil. Thus, the aim of this study was to identify factors associated with musculoskeletal symptoms and heart rate variability (HRV) among cleaners.

**Methods:**

A cross-sectional study conducted at a public higher education institution with 45 outsourced cleaners following approval from the institutional ethics committee. The participants answered a questionnaire addressing sociodemographic, occupational and health data, the Nordic Musculoskeletal Questionnaire, the Physical Activity Questionnaire (work and leisure) and the short version of the Copenhagen Psychosocial Questionnaire. Clinical data (height, body mass, waist-to-hip ratio and blood pressure) and heart rate variability (HRV) were also collected. Logistic and linear regression models were created to identify factors associated with symptoms and HRV.

**Results:**

The sample consisted of women (100%) predominantly older than 50 years of age (44%), without a conjugal life (64%), with three or more children (59%), low educational level (58%) and who worked less than 12 months at the company (87%). Systemic arterial hypertension (23%) was the most reported health problem. The highest frequency of musculoskeletal symptoms was identified in the lower limbs (ankles/feet: 31% in the previous 12 months and 24% in the previous 7 days; knees: 31% in the previous 12 months and 20% in the previous 7 days). Moreover, the workers reported not practicing physical activity during leisure time (84%). Psychosocial aspects indicated health risks for the dimensions “influence at work” (74%), “burnout” (59%) and “stress” (52%). Associations were found between ankle/foot symptoms and body mass index, shoulder symptoms and predictability, and knee symptoms and self-rated health and burnout. HRV indices were associated with age.

**Conclusions:**

This study outlined the profile of female cleaners and identified risk factors. The workers exhibited musculoskeletal symptoms, which were associated with the body mass index and some psychosocial factors. HRV indices were associated with age. Thus, health promotion and prevention measures should be taken to benefit this population of workers.

## Background

Workers who perform cleaning and maintenance activities make up an important sector of the economy in Brazil [1]. Cleaning work does not require highly qualified skills, is performed mainly by women and involves manual tasks with little mechanization [2–6].

The risk factors associated with cleaning work include exposure to chemical agents, falls on slippery surfaces and stairs, working at heights, long working hours, social isolation, lack of autonomy, time pressure, exposure to pathogens, contact with contaminated surfaces and contact with unpleasant odors [5]. Moreover, such workers have dynamic work demands, including intense work pace, frequent upper limb and spine movements, static muscle work, and pulling, pushing, standing and walking activities [2, 3, 5, 7]. Therefore, these individuals may develop health problems related to the musculoskeletal and cardiovascular systems [4, 8–10].

Martarello et al. [9] evaluated hygiene and cleaning workers at a hospital using the Nordic Musculoskeletal Questionnaire; authors identified a high prevalence of shoulder (50%), upper back (43%), neck (37%), and lower back (37%) symptoms. Another prevalence study found highly frequent symptoms in the wrist/hands (42%), shoulder (42%), lower back (38%) and elbows (33%) among cleaning staff at public buildings [11]. Similarly, a study conducted in Sweden [12] identified highly frequent symptoms in the neck/shoulders (49 to 64%) and elbows/hands (41 to 51%).

These workers also have psychosocial risk factors. Some studies have found an association between psychosocial aspects and upper limb musculoskeletal symptoms [13] and arterial hypertension [14] among cleaning staff. Others report high frequencies of hypertension, low cardiorespiratory fitness and cardiovascular risk, which are related to the physical workload of this population [5, 7, 8, 10, 15]. Physical work demands can result in an increased risk of cardiovascular disease and mortality [16, 17], since upper limb activity increases the heart rate and blood pressure [10, 18]. Thus, cleaners are exposed to several cardiovascular risk factors and it is important to evaluate cardiac autonomic modulation in this population.

Heart rate variability (HRV) is used to evaluate autonomic imbalances and cardiovascular disease. It is also used as an indicator of morbidity and mortality [19]. Autonomic imbalance, characterized by a hyperactive sympathetic system and a hypoactive parasympathetic system, is associated with various pathological conditions, including obesity, hypertension and diabetes [19–21]. Although cleaners have risk factors for cardiovascular disease, no studies were found investigating HRV in this population.

A broad approach with the aim of identifying biological, psychological, social and organizational aspects is needed to gain a better understanding of the profile of these workers. Some studies in Brazil have investigated the profile, organizational aspects, working conditions [4, 22–24], work ability [25–27] and musculoskeletal symptoms [6, 9] in cleaners. However, this population is largely overlooked and there is a lack of knowledge regarding their working and health conditions. Such knowledge would enable the proposal of preventive interventions that could benefit large numbers of workers.

Therefore, the aim of the present study was to identify factors associated with musculoskeletal symptoms and HRV among cleaners. Our hypothesis is that the individual (age, body mass index and waist-to-hip ratio) and psychosocial factors are associated with musculoskeletal symptoms and HRV among workers in the cleaning sector.

## Methods

### Type of study

A descriptive cross-sectional study was conducted in accordance with the STROBE (STrengthening the Reporting of OBservational studies in Epidemiology) recommendations (Additional file 1) [28].

### Setting and participants

All outsourced workers of a cleaning company that served a higher education institution were invited to participate in the study. The inclusion criteria were age between 18 to 65 years and a work routine of at least 4 h per day 5 days per week (at least 20 h per week). The exclusion criteria were not being able to participate in the evaluations and being pregnant. This study received approval from the local human research ethics committee (certificate number 56065316.3.0000.5504) and all participants signed a statement of informed consent.

**Fig. 1:**
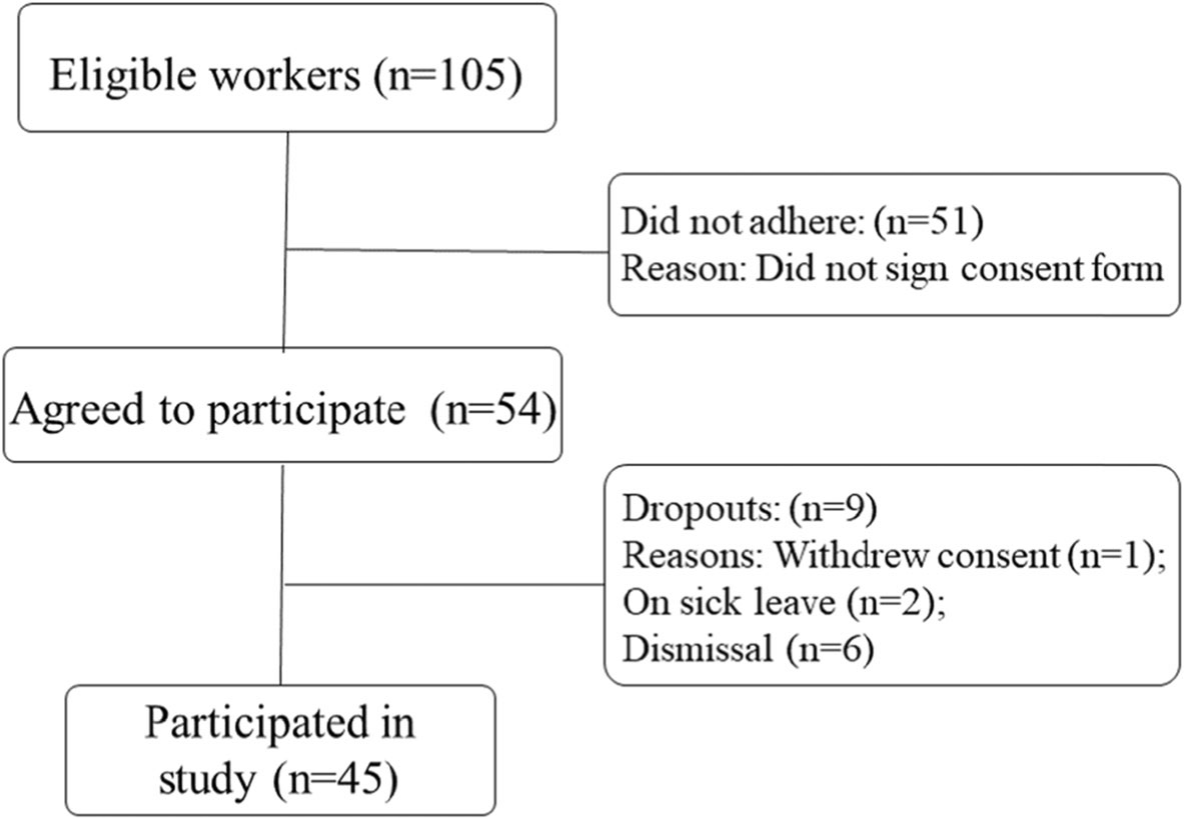
Study flowchart

Among the 105 workers who were employed during the data collection period (January 2017 to July 2018), 45 (43%) agreed to participate and met the inclusion criteria (Fig. 1).

### Instruments

#### Outcomes

The Nordic Musculoskeletal Questionnaire was administered to identify musculoskeletal symptoms (pain, numbness or discomfort) in different body regions in the previous 7 days and 12 months as well as the search for health care and functional limitations due to these symptoms [29]. For each body part the participant answer yes/no about the presence of the symptoms (dichotomous variables).

#### Determinants

A questionnaire was administered addressing demographic (age, sex, marital status and education), occupational (function, time on the job, working hours per day) and general health (smoking, alcohol consumption, health problems, medical treatment and medication use) information (Additional file 2).

The short version of the Copenhagen Psychosocial Questionnaire (COPSOQ-II) was used to evaluate psychosocial aspects of the work [30, 31]. This questionnaire consists of 23 dimensions and 40 questions [31]. The questions are scored on a five-point scale (0 = never, 1 = rarely, 2 = sometimes, 3 = often, 4 = always or 0 = very little, 1 = little, 2 = somewhat, 3 = to a large extent, 4 = to a very large extent). Question 1B is the only item with an inverted score (0 = always, 1 = often, 2 = sometimes, 3 = rarely, 4 = never). The dimensions scores are determined by the sum of the individual items, except for the offensive behavior dimension [30], and range from 0 to 4 points (job satisfaction and health and wellness dimensions) or 0 to 8 points (quantitative demands and trust in management). A color code is also attributed to each dimension: green = favorable; yellow = requires attention; and red = unfavorable [32].

The level of physical activity during work and leisure was investigated using the questionnaire developed by Saltin and Grimby [33], which is composed of two questions, each with four response options. The response options for the question on physical activity at work are 1) predominantly seated, 2) sitting or standing, 3) walking and 4) heavy manual labor. The options for physical activity during leisure time are 1) almost completely inactive (reading, watching TV, movies), 2) physical activity at least 4 h per week (cycling or walking to work), 3) regular activity (running, calisthenics, walking, etc) and 4) regular physical training (sports, running, bodybuilding, etc). For this question, response anchors correspond to sedentary, light, moderate or vigorous physical activity [34].

### Equipment

A digital stadiometer (WISO W721, maximum capacity: 180 kg; precision: 100 g) and tape measure were used to determine the anthropometric data. A digital automatic pressure device (G Tech, Mod. BP3AF1–3) was used to measure blood pressure and a heart rate monitor was used to record the RR intervals of the electrocardiogram (Polar® V800 model).

### Procedures

The evaluation process occurred on two different days. The participants received clarifications regarding the procedures and signed the statement of informed consent on the first day. The questionnaires were administered and the clinical evaluation was performed on the second day. The evaluations were scheduled in advance and the guidelines for the data collection process were provided to the participants. The data were collected at the workplace in a reserved environment in a period of approximately 50 min.

#### Questionnaires

The questionnaires were administered in interview form to ensure the understanding of all items. All procedures were performed at the workplace without any type of burden to the worker.

#### Clinical evaluation

Anthropometric data and blood pressure were collected at rest (sitting). Height, weight, waist circumference and hip circumference were measured with the participants barefoot and wearing light clothing. The body mass index (BMI) was calculated as weight divided by height squared (kg/m^2^) [35, 36]. Waist circumference was measured at the midpoint between the last rib and iliac crest [35, 36]. The waist-to-hip ratio (WHR) was calculated by waist circumference (cm) divided by hip circumference (cm) [37]. After two waist and two hip measurements were performed, means were used to calculate WHR.

Blood pressure (BP) was measured on the left wrist after a five-minute resting period in the seated position. The measurement was made with the palm up and the cuff raised to the heart level. Three consecutive measurements were performed with approximately 30 s rest between each measurement [37].

For the evaluation of HRV, the participants were instructed not to drink alcoholic beverages, take stimulants or smoke in the 24 h prior to the tests, to have a light meal at least 2 h before the tests and not to practice physical exercise other than their routine work and leisure activities. During the clinical evaluation, the participants were instructed to maintain spontaneous breathing. The heart rate monitor was placed on the chest with an elastic strap. The R-R intervals (iRR) of the electrocardiogram were collected at a frequency of 1000 Hz [38]. Readings were performed at rest in both supine and orthostatic standing positions for 10 min.

#### Data processing

The iRR data were transferred to a microcomputer with the FlowSync software. Data processing was performed using the Kubios HRV Analysis software, version 2.0 [39]. Stable sequences with 256 consecutive beats were selected [38]. The analyses were performed using linear models in the time and frequency domains. In the time domain, the RMSSD (square root of the square sum of differences between individual iRR values, divided by the number of iRRs of the selected data series minus one) and SDNN (standard deviation of all iRRs of the data series in the time interval) indices were calculated [38]. The RMSSD index is related to vagal modulation and the SDNN index is related to sympathetic and vagal cardiac modulation.

The HRV data were analyzed in the frequency domain using spectral analysis with the autoregressive method. This method enables the identification of low frequency (LF: 0.04 to 0.15 Hz) and high frequency (HF: 0.15 to 0.40 Hz) components in absolute and normalized units (nu) and the ratio between low and high frequency (LF/HF) [40]. The LF component is related to the joint action of the vagal and sympathetic components. The HF component is related to respiratory modulation and vagal cardiac modulation. The LF/HF ratio reflects sympatovagal balance.

### Data analysis

Descriptive analysis was performed using mean, standard deviation and frequency values to characterize the sample. The HRV indices did not exhibit normal distribution on the Shapiro-Wilk test. Thus, these indices were transformed using the logarithmic function (ln). The paired t-test was applied to log transformed indices for the comparison between the supine and orthostatic standing positions.

Associations between musculoskeletal symptoms in the previous 12 months and the independent variables: age, BMI, WHR and psychosocial aspects were tested using logistic regression analysis. Unadjusted analysis was initially performed between musculoskeletal symptoms and determinants (age, BMI, WHR and psychosocial aspects); associations with *p*-values of less than or equal to 0.20 were included in multivariable analyses [41]. Also, as the number of participants with symptoms was low for most body regions, only the regions with the highest number of cases were considered in this analysis (shoulder, knee and ankle).

Linear regression was applied to evaluate the association between the HRV indices in the supine position and the independent variables: age, BMI, WHR and psychosocial aspects. Unadjusted analysis was initially performed between HRV indices and determinants (age, BMI, WHR and psychosocial aspects); associations with *p*-values of less than or equal to 0.20 were included in multivariable analyses [42]. Data analysis was performed using the SPSS version 25.0 (SPSS Inc., Chicago, IL, USA), with the significance level set at *p* ≤ 0.05.

## Results

The sample consisted entirely of women (100%), 44% of whom were older than 50 years of age. Most did not have a conjugal life (64%), 93% had children and most had three or more children (59%). A total 58% had only up to a complete elementary school. The majority of respondents worked at the company for less than 12 months (87%), with a weekly workload of 45 h [9 h/day 5 days of the week (Monday to Friday)]. Regarding lifestyle, 20% reported smoking, 20% reported using alcohol, 66% reported some health problem, 46% had undergone medical treatment in the previous 3 months and 63% used medications in the previous 2 weeks. The most frequently reported health problem was systemic arterial hypertension (23%). The characterization of the sample is displayed in Table 1.

**Table 1:** Sociodemographic, occupational and health characteristics of cleaning workers (*n* = 45)

Characteristic	% (n)
**Age (years)**	
< than 30 years	8.8 (4)
30–39 years	24.4 (11)
40–49 years	22.2 (10)
> than 50 years	44.4 (20)
**Marital status**	
Single, divorced or widowed	64.4 (29)
Married or living with partner	35.6 (16)
**Children (*n*** **= 44)**	
No	4.4 (2)
Yes	93.3 (42)
**Number of children (%)**	
0	4.5 (2)
1 to 2	36.4 (16)
3 or more	59.1 (26)
**Educational level (%)**	
Illiterate	2.2 (1)
Incomplete elementary school	17.8 (8)
Complete elementary school	37.8 (17)
Incomplete high school	11.1 (5)
Complete high school	28.9 (13)
Incomplete higher education	2.2 (1)
**Time at company in months (%)**	
Up to 12 months	86.7 (39)
More than 12 months	13.3 (6)
**Smoking**^**a**^	
No	79.6 (35)
Yes	20.4 (9)
**Consumption of alcoholic beverages**^**a**^	
No	79.6 (35)
Yes	20.4 (9)
**Current health problem**^**a**^	
No	34.0 (15)
Yes	66.0 (29)
**What health problems?**^**a**^	
Hypertension	22.7 (10)
Diabetes	9.1 (4)
Asthma, bronchitis or pulmonary emphysema	6.9 (3)
Gastrointestinal or hormonal (thyroid)	11.4 (5)
**Medical treatment in previous 3 months**^**a**^	
No	54.5 (24)
Yes	45.5 (20)
**Use of medication in previous 2 weeks**^**a**^	
No	36.3 (16)
Yes	63.6 (28)

^a^ missing data

Table 2 displays the musculoskeletal symptoms experienced by the participants in the previous 12 months, impairment regarding activities of daily living, demands for health care and symptoms in the previous 7 days. The prevalence of symptoms was higher in the ankles/feet and knees, followed by the shoulders and upper back.

**Table 2:** Prevalence of musculoskeletal symptoms in different body regions [% (n)] (*n* = 45)

Body regions	Previous 12 months	Impairment	Demand for care	Previous 7 days
Neck	15.6 (7)	6.7 (3)	8.9 (4)	8.9 (4)
Shoulders	28.9 (13)	6.7 (3)	15.6 (7)	20.0 (9)
Upper back	15.6 (7)	6.7 (3)	6.7 (3)	15.6 (7)
Elbows	4.4 (2)	0 (0)	0 (0)	6.7 (3)
Lower Back	4.4 (2)	2.2 (1)	4.4 (2)	6.7 (3)
Wrists and hands	17.8 (8)	2.2 (1)	4.4 (2)	8.9 (4)
Hip and thighs	6.7 (3)	2.2 (1)	2.2 (1)	6.7 (3)
Knees	31.2 (14)	8.9 (4)	22.2 (10)	20.0 (9)
Ankles and feet	31.2 (14)	2.2 (1)	11.1 (5)	24.4 (11)

Based on the questionnaire developed by Saltin and Grimby [33], 82% of the women considered their physical activity to be in the “walking” category and 84% were sedentary in their leisure time. A small portion was physically active during leisure time (Table 3).

**Table 3:** Levels of physical activity at work and leisure (*n* = 44)

Physical activity	% (n)
Work	
Predominantly sitting	0.0 (0)
Sitting or standing	6.8 (3)
Walking	81.8 (36)
Heavy manual labor	11.4 (5)
Leisure	
Sedentary	84.1 (37)
Light physical activity	9.1 (4)
Moderate physical activity	4.5 (2)
Vigorous physical activity	2.2 (1)

In the evaluation of the psychosocial aspects, the dimensions influence at work (74.1%), burnout (59.3%), stress (51.9%) were the most frequently reported health risk. Unwanted sexual attention and threats of violence (0.0%), and quantitative and emotional work demands, meaning work, physical violence and bullying (7.4%) were the least frequently reported health risk (Table 4).

**Table 4:** Frequency of participants for each dimension of the Copenhagen psychosocial questionnaire (*n* = 27)

Dimensions	Favorable situation	Intermediate risk	Health risk
Influence at work	11.1	14.8	74.1
Burnout	29.6	11.1	59.3
Stress	37.0	11.1	51.9
Work-family conflict	51.1	0.0	48.1
Management/worker trust	51.9	3.7	44.4
Self-rated health	25.9	33.3	40.8
Justice & respect	48.4	14.9	37.0
Work pace	40.9	29.6	29.6
Social support	33.3	40.8	25.9
Appreciation & recognition	51.9	22.2	25.9
Role clarity	51.9	25.9	22.2
Predictability	55.6	22.2	22.2
Leadership quality	59.3	18.5	22.2
New skill development	63.0	18.5	18.5
Commitment to workplace	74.1	14.8	11.1
Job satisfaction	88.9	0.0	11.1
Quantitative work demands	88.9	3.7	7.4
Emotional work demands	70.4	22.2	7.4
Meaningful work	85.1	7.4	7.4
Physical violence	0.0	0.0	7.4
Bullying	0.0	0.0	7.4
Unwanted sexual attention	0.0	0.0	0.0
Threats of violence	0.0	0.0	0.0

The analysis of the clinical data revealed that 2% were underweight, 36% were in the ideal range and 61% were overweight and/or obese. Moreover, the mean WHR was 0.87 (SD = 0.09) and 75% of the women were a high cardiovascular risk (WHR ≥ 0.85).

Mean resting systolic blood pressure was 125 ± 18 mmHg, diastolic blood pressure (DBP) was 81 ± 13 mmHg and heart rate (HR) was 74 ± 9 bpm. Among the 43 workers who participated in this evaluation, 51% had normal BP (SBP ≤ 120 mmHg and/or DBP ≤ 80 mmHg), 33% were pre-hypertensive (SBP 121 to 139 mmHg and/or DBP 81 to 89 mmHg), 14% had Stage 1 hypertension (SBP 140 to 159 mmHg and/or DBP 90 to 99 mmHg) and 2% had Stage 3 hypertension (SBP ≥ 180 mmHg and/or DBP ≥ 110 mmHg).

Table 5 show the HRV indices in the supine and orthostatic standing positions. Statistically significant differences between the positions were found for all variables, except the SDNN index and the LF component.

**Table 5:** Mean (SD), *p*-value* and effect size (dz) for HRV indices (*n* = 40)

HRV indices	Supine	Standing	***P***	Effect size (dz)
iRR (ms)	855.8 (97.2)	738.7 (89.0)	< 0.01	1.61
SDNN (ms)	39.3 (20.6)	39.7 (15.5)		
ln SDNN	1.54 (0.19)	1.56 (0.19)	0.72	0.09
RMSSD (ms)	29.6 (22.8)	20.8 (11.5)		
ln RMSSD	1.37 (0.28)	1.24 (0.25)	< 0.01	0.56
LF (ms^2^/Hz)	544.2 (621.6)	746.2 (610.9)		
ln LF	2.56 (0.38)	2.69 (0.44)	0.07	0.28
HF (ms^2^/Hz)	473.0 (817.7)	204.2 (225.0)		
ln HF	2.26 (0.59)	2.02 (0.57)	< 0.01	0.49
LF/HF	2.68 (2.08)	6.19 (4.50)		
ln LF/HF	0.29 (0.35)	0.67 (0.35)	< 0.01	1.38

*SD* Standard deviation, *HRV* Heart rate variability, *iRR* RR Interval, *SDNN* Standard deviation of all iRR of data series in time interval, *RMSSD* Square root of mean square differences of successive RRs, *LF* Low frequency, *HF* High frequency, *LF/HF* Sympatovagal balance

*paired t-test

Shoulder symptoms were significantly associated with predictability. Knee symptoms were significantly associated with self-rated health and burnout. Ankle/foot symptoms were associated with BMI (Table 6).

**Table 6:** Simple and multiple logistic regression analysis to determine the independent predictors of shoulder, knee and ankle/foot symptoms in the previous 12 months

Variables	Simple OR (95% CI)	Multiple OR (95% CI)
**Shoulder**		
Body mass index	1.11 (0.99 to 1.25)	1.16 (0.82 to 1.65)
Influence at work	1.34 (0.90 to 1.98)	1.20 (0.56 to 2.58)
New skill development	1.39 (0.86 to 2.25)	2.29 (0.84 to 6.19)
Predictability	0.71 (0.49 to 1.03)	**0.62 (0.40 to 0.96)**
Appreciation & recognition	0.75 (0.52 to 1.06)	0.89 (0.40 to 1.95)
Management/worker trust	0.80 (0.57 to 1.13)	1.20 (0.60 to 2.36)
Justice & respect	0.68 (0.45 to 1.02)	0.55 (0.23 to 1.34)
Burnout	1.30 (0.88 to 1.93)	0.88 (0.49 to 1.59)
**Knee**		
Quantitative work demands	2.12 (1.06 to 4.24)	4.31 (0.03 to 6.33)
Work pace	1.26 (0.89 to 1.77)	1.84 (0.50 to 6.72)
Emotional work demands	0.69 (0.41 to 1.14)	0.23 (0.02 to 2.21)
Influence at work	0.71 (0.45 to 1.13)	1.31 (0.50 to 3.43)
Self-rated health	0.39 (0.15 to 0.99)	**0.17 (0.03 to 0.88)**
Burnout	0.65 (0.41 to 1.04)	**0.51 (0.27 to 0.98)**
Stress	0.74 (0.51 to 1.07)	1.32 (0.31 to 5.64)
**Ankle/foot**		
Body mass index	1.19 (1.03 to 1.36)	**1.30 (1.02 to 1.60)**
Role clarity	1.43 (0.88 to 2.32)	1.78 (0.90 to 3.50)
Work-family conflict	1.27 (0.88 to 1.84)	1.51 (0.87 to 2.61)

Three individual risk factors and 19 occupational exposures (mechanical, psychosocial) were examined in unadjusted models. Only those models with an association of *p* < 0.20 in unadjusted models between symptoms and determinants were included in the table

Associations were found between age and the SDNN, RMSSD, HF, LF and LF/HF indices in the multiple regression (Table 7).

**Table 7:** Simple and multiple linear regression analysis to determine the independent predictors the HRV indices in the supine position

Variables	Simple regression B (95% CI)	Multiple regression B (95% CI)
**iRR**		
Quantitative work demands	−16.9 (−38.8 to 5.0)	−12.9 (−37.5 to 11.6)
Role clarity	12.6 (−6.39 to 31.5)	7.7 (−13.2 to 28.7)
**ln SDNN**		
Age	−0.01 (−0.01 to 0.00)	**−0.01 (−0.02 to − 0.01)**
Waist-hip ratio	−0.74 (− 1.45 to −0.02)	0.45 (−0.75 to 1.66)
Work pace	0.02 (−0.01 to 0.06)	0.09 (−0.02 to 0.04)
**ln RMSSD**		
Age	−0.01 (− 0.02 to 0.00)	**− 0.01 (− 0.02 to 0.00)**
Waist-hip ratio	− 1.40 (−2.39 to − 0.41)	− 0.72 (− 1.77 to 0.33)
**ln LF**		
Age	− 0.02 (− 0.04 to − 0.01)	**−0.02 (− 0.04 to − 0.01)**
Waist-hip ratio	−1.27 (−2.71 to 0.16)	0.85 (− 1.58 to 3.28)
Stress	0.05 (− 0.02 to 0.12)	0.01 (− 0.05 to 0.07)
**ln HF**		
Age	− 0.03 (− 0.04 to − 0.01)	**−0.03 (− 0.04 to − 0.01)**
Waist-hip ratio	−1.27 (− 2.71 to 0.16)	−1.24 (− 3.56 to 1.06)
**ln LF/HF**		
Age	0.01 (0.00 to 0.02)	**0.01 (0.00 to 0.02**)
Waist-hip ratio	1.33 (0.05 to 2.62)	−0.53 (− 3.01 to 1.95)
Appreciation & recognition	0.04 (−0.02 to 0.10)	0.03 (− 0.02 to 0.09)

Three individual risk factors and 19 occupational exposures (mechanical, psychosocial) were examined in unadjusted models. Only those models with an association of *p* < 0.20 in unadjusted models between HRV indices and determinants were included in the table

*iRR* Interval RR, *SDNN* Standard deviation of all iRRs of data series in time interval, *RMSSD* Square root of mean square differences of successive RRs, *LF* Low frequency, *HF* High frequency, *LF/HF* Sympatovagal balance

## Discussion

The present study describes the sociodemographic, occupational and health profile of cleaners. The cleaning work was performed by middle-aged women who lived without a partner, had three or more children and had a low educational level. Considerable turnover was found in the sector and the most commonly reported health problem was hypertension.

These findings are consistent with data from a study conducted in Brazil on the profile of outsourced cleaning workers at a university hospital. The study showed that 74% of the workers were female, 94% did not have a complete high school education, 44% had worked for less than a year at the company and 36% had diagnosed diseases, the most prevalent of which were respiratory (29%), cardiovascular (26%) and musculoskeletal (10%) diseases [4].

The regions most affected by musculoskeletal symptoms among the cleaning workers were the shoulders, knees and ankles/feet. Silva et al. [6] evaluated cleaning workers at a higher education institution and found that the most affected parts of the body were the lower limbs (17%), followed by the neck, low back and shoulders (16%). These findings may be explained by the fact that such work requires long hours of walking and remaining in the standing position. Other studies on workers in the cleaning sector of hotels, hospitals and public buildings report that the regions with the highest prevalence of symptoms were the shoulders, thoracic spine, neck and lumbar spine [9, 11, 12, 43]. The differences between the present results and findings described in the studies cited may be due to the characteristics of the workplace evaluated in this study, which is a university with a very large campus and buildings distant from each other, requiring workers to walk long distances wearing safety shoes. Several studies have associated the use of such footwear with symptoms in the ankles/feet [44, 45]. Footwear is seen by companies as protective equipment for the worker, but does not prevent musculoskeletal disorders and the design can directly affect plantar pressure, comfort, fatigue levels and muscle activation [46, 47].

An association was also found between the body mass index and symptoms in the ankle/foot region. Studies have shown that an increasing body mass index over time is a predictive factor for the development of symptoms in women, such as pain in the ankles/feet [48–50]. A high body mass index can lead to an excessive load on the structures of the foot joints (generally from adipose tissue), resulting in symptoms in this region [50].

Shoulder symptoms were associated with predictability and knee symptoms were associated with self-rated health and burnout. Some studies have investigated the risk factors for musculoskeletal symptoms and have shown associations with psychosocial factors, with low social support from colleagues and/or superiors in the workplace, as well as self-rated health and burnout increasing the risk of symptom development [51–54].

With regards to psychosocial aspects, women in the cleaning sector often work alone in dispersed areas, receiving little social support from supervisors and workmates, which can result in psychosocial stress [5]. Moreover, low decision-making power at the workplace can lead to the development of mental disorders, such as stress, burnout, anxiety and depression [55, 56]. Physical violence and bullying at the workplace was another important aspect reported by the cleaning workers and can have a negative impact on mental wellbeing. The severity of this impact can increase due to psychological distress and little social support from supervisors [57].

The level of physical activity at work indicated that the majority of workers have a high work demand (“walking”) and most do not perform physical activity during their leisure time [33]. Korshøj et al. [7] found that cleaning workers walked more than 20,000 steps per day, but most had low cardiorespiratory fitness, possibly due to the low intensity of physical activity performed at work. Studies on occupational and leisure physical activity indicate that extensive walking during work can lead to muscle fatigue-induced lower limb symptoms, resulting in a sedentary lifestyle during leisure time [58, 59].

The clinical results are in agreement with data described by Jørgensen et al. [60], who also found high frequencies of overweight, hypertension and other health problems in immigrant cleaning workers when compared to Danish workers. According to Korshøj et al. [10], the increased risk of cardiovascular disease in cleaners may be mediated by increased blood pressure due to the prolonged duration of physical activity at work.

Another important finding was the HRV indices recorded in the supine and orthostatic standing positions. The LF component, which reflects sympathetic and parasympathetic modulation, was higher than the reference values published after a 1996 Task Force report and quantitative systematic review, designed to quantify reference ranges for short-term measures of HRV in healthy adult populations [61]. In addition, HRV indices are reduced in both the supine and orthostatic standing positions. This shows the imbalance of the cardiac autonomic system that can be related to the development of cardiovascular diseases [19–21]. HRV indices were also inversely associated with age, indicating that HRV indices in the time and frequency domains decrease and the LF/HF ratio increases with the increase in age. Similar findings have been reported in other populations [62–65]. These findings, together with the data on hypertension, a sedentary lifestyle and the WHR, indicate an increase in cardiovascular risk in this population.

### Limitations and perspectives

The present study has some limitations that should be considered. The evaluation was carried out at the workplace, which may have led to a lack of concentration on the responses and increased concern, since the work was interrupted. Another limitation is the cross-sectional design which does not allow causal inferences. Cross-sectional studies measure exposure and outcome at the same time and it is often difficult to establish a temporal relationship between exposure and outcome, that is, whether exposure or disease occurs first. Future studies should include participants’ follow up in a longitudinal design, including objective measurements during work. In addition, the low sample size may have hampered the associations between musculoskeletal symptoms and psychosocial factors. Also, chemical exposures were not evaluated in the study, which could be a potential confounder in the association between HRV and the explanatory variables.

However, some points should be highlighted, such as: the STROBE guidelines were followed, the surveys were administered through interviews instead of self-administered and 43% agreed to participate in the study. Still, the results of the present study are limited to workers in the outsourced cleaning sector and, therefore, cannot be generalized to other worker populations.

Despite these limitations, the present findings indicate prospects for future studies with this population. Interventions should be proposed that include means of transportation, pauses, changes of position (standing to sitting) as well as adequate shoes, insoles and compression socks to reduce fatigue and symptoms in the lower limbs [66]. It would also be important to implement an intervention program focused on the practice of aerobic exercise in the work environment. Several studies have reported the positive effects of aerobic exercise for the improvement of cardiovascular health and musculoskeletal symptoms [67–69].

## Conclusions

This study outlined the profile and identified risk factors in cleaning workers. The women had musculoskeletal symptoms in the ankles/feet, which were associated with the BMI. HRV indices were associated with age. Thus, health promotion and prevention measures should be taken to benefit this population of workers.

## Supplementary information

**Additional file 1.** STROBE Checklist.

**Additional file 2.** Sociodemographic and Health Questionnaire.

## Data Availability

The datasets used during the present study are available from the corresponding author upon request.

## Abbreviations

HRV: Heart rate variability
BMI: Body mass index
WHR: Waist-to-hip ratio
BP: Blood pressure
iRR: RR interval
SDNN: Standard deviation of all iRRs of data series in time interval
RMSSD: Square root of mean square differences of successive RRs
LF: Low frequency
HF: High frequency
LF/HF: Sympatovagal balance
