# Supplementary material for: Factors associated with musculoskeletal symptoms and heart rate variability among cleaners – cross-sectional study
Source: BMC Public Health. 2020 May 24;20:774. doi: 10.1186/s12889-020-08928-7 (PMC7247127; doi:10.1186/s12889-020-08928-7)
Supplement: Supplementary file 1 — Additional file 1. STROBE Checklist. [file 12889_2020_8928_MOESM1_ESM.doc]

STROBE Statement—Checklist of items that should be included in reports of ***cross-sectional studies***

|  | Item No | Recommendation |
| --- | --- | --- |
| **Title and abstract** | 1 | (*a*) Indicate the study’s design with a commonly used term in the title or the abstract  **Authors' response:** *The study design is indicated in the “title and abstract section” by the term "cross-sectional study".* |
|  |  | (*b*) Provide in the abstract an informative and balanced summary of what was done and what was found  **Authors' response:** *We seek to make a clear and concise summary of all that has been done and as a result found in this study.* |
| Introduction | | |
| Background/rationale | 2 | Explain the scientific background and rationale for the investigation being reported  **Authors' response:** *In the “introduction section”, we briefly present the cleaning work and the risks involved with this type of work, as well as musculoskeletal and cardiovascular health problems (section “Background; pages 3,4 and 5).* |
| Objectives | 3 | State specific objectives, including any prespecified hypotheses  **Authors' response:** *The aim the study was to identify factors associated with musculoskeletal symptoms and heart rate variability (HRV) among outsourced workers in the cleaning sector of a higher education institution.* *The hypothesis of the study is that the individual factors (age, body mass index and waist-to-hip ratio) and psychosocial factors are associated with musculoskeletal symptoms and heart rate variability in workers in the cleaning sector (section “Background; page 5).* |
| Methods | | |
| Study design | 4 | Present key elements of study design early in the paper  **Authors' response:** *An observational cross-sectional study was conducted in accordance with the STROBE (section* *Methods; page 5).* |
| Setting | 5 | Describe the setting, locations, and relevant dates, including periods of recruitment, exposure, follow-up, and data collection  **Authors' response:** *All outsourced workers of a cleaning company that served a higher education institution were invited to participate in the study. Among the 105 workers who were employed during the data collection period (January 2017 to July 2018). (subsection “Setting and participants”; page 5 and 6).* |
| Participants | 6 | (*a*) Give the eligibility criteria, and the sources and methods of selection of participants  **Authors' response:** *The study included workers* *with age between 18 to 65 years and a work routine of at least four hours per day five days per week (at least 20 hours per week). The exclusion criteria were not being able to participate in the evaluations and being pregnant. 45 (43%) agreed to participate in the study and met the inclusion criteria. This study received approval from the local human research ethics committee (certificate number: 56065316.3.0000.5504) and all participants signed a statement of informed consent (subsection “Setting and participants”; page 5 and 6).* |
| Variables | 7 | Clearly define all outcomes, exposures, predictors, potential confounders, and effect modifiers. Give diagnostic criteria, if applicable |
| Data sources/ measurement | 8* | For each variable of interest, give sources of data and details of methods of assessment (measurement). Describe comparability of assessment methods if there is more than one group  **Authors' response:** *The participants answered a questionnaire addressing sociodemographic, occupational and health data, the Nordic Musculoskeletal Questionnaire, the Physical Activity Questionnaire (work and leisure) and the short version of the Copenhagen Psychosocial Questionnaire. Clinical data (height, body mass, waist-to-hip ratio and blood pressure) and heart rate variability (HRV) were also collected (pages 6, 7, 8 and 9).* |
| Bias | 9 | Describe any efforts to address potential sources of bias |
| Study size | 10 | Explain how the study size was arrived at  **Authors' response:** *The study sample consists of a convenience sample. All participants who met the inclusion criteria and agreed to participate were included in the study.* |
| Quantitative variables | 11 | Explain how quantitative variables were handled in the analyses. If applicable, describe which groupings were chosen and why.  **Authors' response:** *The quantitative variables included in the present study were: age, BMI, WHR, HRV. Musculoskeletal symptoms consist of a qualitative variable that has been decoded for statistical analysis. Descriptive analysis was applied to the quantitative data (mean and standard deviation).* |
| Statistical methods | 12 | (*a*) Describe all statistical methods, including those used to control for confounding  **Authors' response:** *Descriptive analysis was performed using mean, standard deviation and frequency values to characterize the sample. The HRV indices did not exhibit normal distribution on the Shapiro Wilk test. Thus, these indices were transformed using the logarithmic function (ln). The paired t-test was applied to log transformed indices for the comparison between the supine and standing positions.*  *Associations between musculoskeletal symptoms in the previous 12 months and age, BMI, WHR and psychosocial aspects were tested using logistic regression analysis. As the number of participants with symptoms was low for most body regions, only the regions with the highest number of cases were considered in this analysis (shoulder, knee and ankle). Simple linear regression was applied to evaluate the association between the HRV indices in the supine position and age, BMI, WHR and psychosocial aspects. Each of the factors of interest was included in the regression model separately (enter method). Data analysis was performed using the SPSS version 25.0 (SPSS Inc, Chicago, IL, USA), with the significance level set at 5%.* |
|  |  | (*b*) Describe any methods used to examine subgroups and interactions  Not applicable |
|  |  | (*c*) Explain how missing data were addressed  Not applicable |
|  |  | (*d*) If applicable, describe analytical methods taking account of sampling strategy  Not applicable |
|  |  | (*e*) Describe any sensitivity analyses  Not applicable |
| Results | | |
| Participants | 13* | (a) Report numbers of individuals at each stage of study—eg numbers potentially eligible, examined for eligibility, confirmed eligible, included in the study, completing follow-up, and analysed  **Authors' response:** *Eligible work (n=105), Did not adhere (n=51), Agreed to participate (n=54) and Participated in study (n=45).* |
|  |  | (b) Give reasons for non-participation at each stage  **Authors' response:** *Did not adhere (n=51) – Reason: Did not sign consent form*  *Dropouts (n=9)*  *Reasons: Withdrew consent (n=1)*  *On sick leave (n=2)*  *Dismissal (n=6)* |
|  |  | (c) Consider use of a flow diagram  **Authors' response:** *Figure 1 of the article.* |
| Descriptive data | 14* | (a) Give characteristics of study participants (eg demographic, clinical, social) and information on exposures and potential confounders  **Authors' response:** *The sample consisted entirely of women (100%), 44% of whom were older than 50 years of age. Most did not have a conjugal life (64%), 93% had children and most had three or more children (59%). A total 58% had only up to a complete elementary school. The majority of respondents worked at the company for less than 12 months (87%), with a weekly workload of 45 hours [nine hours/day five days of the week (Monday to Friday)]. Regarding lifestyle, 20% reported smoking, 20% reported using alcohol, 66% reported some health problem, 46% had undergone medical treatment in the previous three months and 63% used medications in the previous two weeks. The most frequently reported health problem was systemic arterial hypertension (23%). The characterization of the sample is displayed in Table 1.* |
|  |  | (b) Indicate number of participants with missing data for each variable of interest  **Authors' response:** *Tables 1 to 5 in the article.* |
| Outcome data | 15* | Report numbers of outcome events or summary measures |
| Main results | 16 | (*a*) Give unadjusted estimates and, if applicable, confounder-adjusted estimates and their precision (eg, 95% confidence interval). Make clear which confounders were adjusted for and why they were included  **Authors' response:** *An unadjusted model was used for simple linear and logistic regression analyses and their precision was reported in the results section.* |
|  |  | (*b*) Report category boundaries when continuous variables were categorized  Not applicable |
|  |  | (*c*) If relevant, consider translating estimates of relative risk into absolute risk for a meaningful time period  Not applicable |
| Other analyses | 17 | Report other analyses done—eg analyses of subgroups and interactions, and sensitivity analyses  Not applicable |
| Discussion | | |
| Key results | 18 | Summarise key results with reference to study objectives  **Authors' response:** *The sample consisted of women (100%) predominantly older than 50 years of age (44%), without a conjugal life (64%), with three or more children (59%), a low educational level (58%) and who worked less than 12 months at the company (87%). Systemic arterial hypertension (23%) was the most reported health problem. The highest frequency of musculoskeletal symptoms was identified in the lower limbs (ankles/feet: 31% in the previous 12 months and 24% in the previous seven days; knees: 31% in the previous 12 months and 20% in the previous seven days). Moreover, the workers reported not practicing physical activity during leisure time (84%). Psychosocial aspects indicated health risks for the items “influence at work” (74%), “health and wellness” (41%), “burnout” (59%), “stress” (52%), “physical violence” (7%), “bullying” (7%) and “social support” (41%). Associations were found between symptoms in the ankle/foot and the body mass index as well as between HRV indexes and both age and the waist-to-hip ratio.* |
| Limitations | 19 | Discuss limitations of the study, taking into account sources of potential bias or imprecision. Discuss both direction and magnitude of any potential bias  **Authors' response:** *The present study has some limitations that should be considered. The evaluation was carried out at the workplace, which may have led to a lack of concentration on the responses and increased concern, since the work was interrupted. Moreover, fear of participating in the study may have occurred, as 48% did not agree to participate.* |
| Interpretation | 20 | Give a cautious overall interpretation of results considering objectives, limitations, multiplicity of analyses, results from similar studies, and other relevant evidence  **Authors' response***: This study outlined the profile of female cleaning workers and identified the risk factors. The workers exhibited musculoskeletal symptoms in the ankles/feet, which were associated with the body mass index. HRV indices were associated with both age and the waist-to-hip ratio. Thus, health promotion and prevention measures should be taken to benefit this population of workers*. |
| Generalisability | 21 | Discuss the generalisability (external validity) of the study results  **Authors' response:** *The results of the present study are limited to workers in the outsourced cleaning sector and, therefore, cannot be generalized to other worker populations.* |
| Other information | | |
| Funding | 22 | Give the source of funding and the role of the funders for the present study and, if applicable, for the original study on which the present article is based  Not applicable. |

*Give information separately for exposed and unexposed groups.

**Note:** An Explanation and Elaboration article discusses each checklist item and gives methodological background and published examples of transparent reporting. The STROBE checklist is best used in conjunction with this article (freely available on the Web sites of PLoS Medicine at http://www.plosmedicine.org/, Annals of Internal Medicine at http://www.annals.org/, and Epidemiology at http://www.epidem.com/). Information on the STROBE Initiative is available at www.strobe-statement.org.
