# Supplementary material for: Factors associated with musculoskeletal symptoms and heart rate variability among cleaners – cross-sectional study
Source: BMC Public Health. 2020 May 24;20:774. doi: 10.1186/s12889-020-08928-7 (PMC7247127; doi:10.1186/s12889-020-08928-7)
Supplement: Supplementary file 2 — Additional file 2. Sociodemographic and Health Questionnaire. [file 12889_2020_8928_MOESM2_ESM.docx]

**Sociodemographic and Health Questionnaire**

Name: _________________________________________________________________ Date: ______________ Mobile: _________________ e-mail: _____________________ Function: _________________________ Place/building: _________________________

Time in position: ___________________ Total hours worked per day: ______________

Age: ________ Sex: ( ) female ( ) male

Education:

( ) incomplete elementary school

( ) complete elementary school

( ) incomplete high school

( ) complete high school

( ) incomplete graduation

( ) complete graduation

Marital Status:

( ) single

( ) married

( ) widowed

( ) divorced

( ) other? _____________________________

Do you have children? ( ) no ( ) yes How many? ____ Ages: __________

Do you smoke? ( ) no ( ) yes How many cigarettes per day? __________

( ) ex-smoker For how long? ______________

Do you consume alcoholic beverages? ( ) no ( ) yes

How many times a week? _______ How much? __________________

Do you practice physical activity? ( ) no ( ) yes

What activity? ____________________ How many times a week? _________________

Have you suffered any trauma (falls, accidents) in the last 2 weeks? ( ) no ( ) yes

Do you have any health problems? ( ) no ( ) yes Which one? ____________________

Did you have health problem in the past? ( ) no ( ) yes Which one? _______________

Do you have high blood pressure (160mmHg/100mmHg)? ( ) no ( ) yes

Do you have angina pectoris? ( ) no ( ) yes

Do you have a pacemaker in your heart? ( ) no ( ) yes

Do you have diabetes? ( ) no ( ) yes

Do you have labyrinthitis? ( ) no ( ) yes

Do you feel dizzy? ( ) no ( ) yes

Do you have the flu or cold? ( ) no ( ) yes

Do you have a herniated disc? ( ) no ( ) yes

Did you had any medical treatment in the last 3 months? ( ) no ( ) yes For what? ____

Do you feel pain in your body? ( ) no ( ) yes

Have you used or are using any medication in the last 2 weeks? ( ) no ( ) yes Which one? _____________________________

Have you had any surgery in the last year? ( ) no ( ) yes Reason: __________________ Are you pregnant? ( ) no ( ) yes

Thank you for your participation!
